# Quantitative Analysis of a Program Director Panel for International Medical Graduates Applying to Physical Medicine and Rehabilitation Residency: A Pilot Study on Self-Perceived Knowledge and Confidence

**DOI:** 10.7759/cureus.106432

**Published:** 2026-04-04

**Authors:** Hsuan Chou, Sojeong Mun, Paul Abboud, I-Ling Chou, Jennifer Zhao, Xiaoyu Pan, Hunter Soleymani, Hana Azizi

**Affiliations:** 1 Public and Ecosystem Health, Cornell University, Ithaca, USA; 2 Physical Medicine and Rehabilitation, Hallym University College of Medicine, Chuncheon, KOR; 3 Anesthesiology and Critical Care Medicine, University of New Mexico School of Medicine, Albuquerque, USA; 4 Physical Medicine and Rehabilitation, China Medical University College of Medicine, Taichung, TWN; 5 Physical Medicine and Rehabilitation, University of Illinois Chicago College of Medicine, Chicago, USA; 6 Physical Medicine and Rehabilitation, MetroWest Medical Center, Framingham, USA; 7 Physical Medicine and Rehabilitation, Mayo Clinic, Rochester, USA; 8 Rehabilitation and Regenerative Medicine, Columbia University Vagelos College of Physicians and Surgeons, New York, USA

**Keywords:** international medical graduates (imgs), medical residency, online medical education, physical medicine and rehabilitation, residency program director

## Abstract

International medical graduates (IMGs) hold a growing number of positions in the physical medicine and rehabilitation (PM&R) workforce; however, many IMGs continue to encounter notable challenges in navigating the United States residency application process. Accordingly, this study organized a one-hour, program director (PD)-led webinar to evaluate the impact of this educational event on attendees’ self-perceived knowledge and confidence of seven key domains, including: (i) their baseline understanding of the PM&R residency program, (ii) writing a personal statement, (iii) building a curriculum vitae and arranging geographic preferences, (iv) communication for letters of recommendation and standard letters of evaluation, (v) utilization of program signaling, (vi) Interview and post-interview communication, and (vii) Other IMG-specific considerations. By comparing pre- and post-session questionnaire results, this retrospective study found statistically significant improvements in IMGs’ self-perceived knowledge and confidence across all seven domains after attending this PD-led panel. Moreover, the overall satisfaction was high, and attendees reported that the panel was well-structured, relevant, and engaging. Accordingly, these findings highlight the importance of direct PD engagement in IMG-focused PMR education and the need for continued research into the specific challenges encountered by IMGs pursuing a PM&R residency application in the United States.

## Introduction

Physical medicine and rehabilitation (PM&R) has experienced substantial growth and rising interest among residency applicants over the past decade. According to data from the National Resident Matching Program (NRMP), used with permission granted by the NRMP, the number of PM&R residency positions increased by 26%, whereas the number of applicants increased by 37% between 2010 and 2020 [1]. This growth in applicants was accompanied by higher academic qualifications among matched candidates, including United States (US) Medical Licensing Examination (USMLE) Step scores, as well as research and volunteer experiences [1]. While this trend shows the expanding appeal of the field, it also indicates an increasing competitiveness in the selection process.

International medical graduates (IMGs) now make up 34% of all active applicants, reflecting their growing presence in the applicant pool of all specialties [2]. Nevertheless, their match rates into PM&R are relatively low. In the 2024-2025 application cycle, US citizen IMGs filled 4.2% of Post-Graduate Year (PGY)-1 and 3.9% of PGY-2 positions, and non-US citizen IMGs filled 3.8% and 2.4% of positions, respectively [2]. This is notable given that IMGs comprise nearly one-quarter of the actively practicing US physician workforce [3].

PM&R has increasingly focused on diversity and inclusion, and many programs acknowledge the value that internationally trained physicians can bring to the field [4]. However, IMGs often encounter informational barriers, including unfamiliarity with US medical education, limited specialty-specific advising, and fewer opportunities for targeted mentorship. Consequently, guidance for essential components of the application process, such as writing a personal statement (PS), building a curriculum vitae (CV), sending signals to programs, and preparing for interviews, may remain insufficient.

Program directors (PDs) play a central role in residency selection and are well-positioned to clarify expectations and provide targeted guidance. However, structured PD-led opportunities specifically designed for IMG applicants remain limited, and few studies have evaluated their impact within PM&R. This pilot study addresses this gap by evaluating the effect of a one-hour interactive session led by PDs on IMG applicants’ self-perceived knowledge and confidence in core application domains. The study also examines participant satisfaction with the educational experience provided by the panel. 

A part of the abstract was presented as a poster at the 2026 Annual Meeting of the Association of Academic Physiatrists on February 19, 2026.

## Materials and methods

This was a single-group pre-post educational study that collected data retrospectively from a one-hour virtual panel completed on July 30, 2025. This research protocol was submitted to the Institutional Review Board at Columbia University (protocol number: ACYY0062) and was determined not to be human subjects research.

Study process

The workshop invited three current US PM&R PDs to answer common questions regarding the application process. The participants were recruited to join the panel by sharing a digital flyer across different social media platforms, including Instagram (Meta Platforms, Inc., Menlo Park, California, US), Reddi (Reddit Inc., San Francisco, California, US), WhatsApp (Meta Platforms, Inc.), X (X Corp., Bastrop, Texas, US), Discord (Discord Inc., San Francisco, California, US), and USMLEKOREA (http://www.usmlekorea.com).

The participants were required to complete anonymous pre- and post-surveys on their improvement in knowledge and confidence in the PM&R residency application process. The organizers of the panel collected questions from a pre-webinar survey by developing a 27-item survey, including seven domains as follows: (i) Baseline understanding of the PM&R residency program, (ii) Writing a PS, (iii) Writing a CV and arranging geographic preferences, (iv) Communication for letters of recommendation (LOR) and standard letters of evaluation (SLOE), (v) Utilization of program signaling, (vi) Interview & post-interview communication, and (vii) Other IMG-specific considerations. Each domain contained four questions, except the first one, which contained three questions. All items were rated on a five-point Likert scale to evaluate both participants’ self-perceived knowledge and confidence (see Appendices).

Study population

Participants were eligible if they (i) identified as IMGs, defined as individuals who completed their medical education outside the US or Canada, (ii) expressed interest in pursuing a residency or career in PM&R, (iii) were able to understand and respond to the survey in English, and (iv) were able to fill out both pre- and post- surveys. Participants were excluded if they (i) were not international medical graduates, (ii) submitted incomplete survey responses, (iii) provided duplicate responses, or (iv) were unable to fill out both pre- and post- surveys.

Data analysis

Demographics were demonstrated using frequencies, percentages, and means with 95% confidence intervals (CIs), and the regions were presented using the United Nations geoscheme [5]. A bar chart and forest plot demonstrated the average score of the seven domains. The statistical analysis includes the reliability of the survey, paired test for pre- and post-surveys, and satisfaction. The reliability test using Cronbach’s alpha (α) was conducted across the seven domains to ensure internal consistency within each domain, and an α ≥ 0.70 was considered reliable [6]. All demographics, 27-item scores, and average domain scores were assessed using a normality test (Shapiro-Wilk test) to confirm if the data were normally distributed. Paired t-tests were used to analyze normally distributed data, and Wilcoxon signed-rank tests were utilized to analyze non-normally distributed data. To control type 1 error due to multiple comparisons across domains, the Holm correction was utilized to correct significance. Lastly, all six satisfaction questions were measured separately on a five-point scale. The satisfaction questions include the flow or structure, time for questioning and answering (Q&A), relevant level of content, engagement level, recommendation level to others, and overall satisfaction with this event. This satisfaction survey also used a five-point Likert scale and was compared to a neutral median of three in a one-sample statistical test. All statistics with a P < 0.01 in this study were considered significant.

## Results

A total of 105 individuals registered for the panel through the provided pre-survey link. Among these, 33 attended the webinar and provided feedback, and 31 participants (29.5%) completed both anonymous pre- and post-surveys, generating effective paired data for analysis.

Demographics

Table 1 summarizes the demographics of the participants. The analytic sample (n = 31) consisted of 15 men (48.4%) and 16 women (51.6%), with a mean age of 30.67 years (95%CI: 28.80-32.54; range 25-45). Most respondents identified as Asian (64.5%), while 22.6% reported White, 9.7% Hispanic, and 3.2% Black or African American backgrounds. Citizenships were diverse with the following regions: East Asia (35.5%), North America (22.6%), Western Asia (9.7%), Southern Asia (9.7%), Central America (6.5%), Eastern Europe (6.5%), South-eastern Asia (6.5%), and Southern Europe (3.2%). Regions of medical training backgrounds mirrored this diversity, with the following regions: Eastern Asia (41.9%), Caribbean (16.1%), Western Asia (9.7%), South-eastern Asia (9.7%), Southern Asia (6.5%), Central America (6.5%), Eastern Europe (6.5%), and Southern Europe (3.2%).

**Table 1:** Demographics (N = 31) * Citizenship: Eastern Asia Subregion: Taiwan (n=5), China (n=4), and South Korea (n=2); Northern America Subregion: United States (n=5) and Canada (n=2); Western Asia Subregion: Turkey (n=2) and Lebanon (n=1); Southern Asia Subregion: India (n=2) and Nepal (n=1); Central America Subregion: Guatemala (n=1) and Mexico (n=1); Eastern Europe Subregion: Belarus (n=1) and Russia (n=1); South-eastern Asia Subregion: Malaysia (n=1) and Thailand (n=1); Southern Europe Subregion: Portugal (n=1) ** Region of Medical School: Eastern Asia Subregion: Taiwan (n=6), China (n=5), and South Korea (n=2); Caribbean Subregion: Sint Maarten (n=2), Aruba (n=1), Cuba (n=1), and Dominican Republic (n=1); Western Asia Subregion: Turkey (n=2) and Lebanon (n=1); South-eastern Asia Subregion: Malaysia (n=1), Philippines (n=1), and Thailand (n=1); Southern Asia Subregion: India (n=1) and Nepal (n=1); Central America Subregion: Guatemala (n=1) and Mexico (n=1); Eastern Europe Subregion: Poland (n=1) and Russia (n=1); Southern Europe Subregion: Spain (n=1) USCE: United States clinical experience, USMLE: United States Medical Licensing Examination

Characteristics			Frequency	Percentage
Gender	Male		15	48.4%
	Female		16	51.6%
Age (years), mean±SD (range)			30.67±4.43 (25-45)	
Race	Asian (Far East, Southeast Asia, Indian)		20	64.5%
	White (Europe, Middle East, North Africa)		7	22.6%
	Hispanic (of any race)		3	9.7%
	Black or African American (African, West Indian, Caribbean)		1	3.2%
Citizenship^*^	Eastern Asia		11	35.5%
	Northern America		7	22.6%
	Western Asia		3	9.7%
	Southern Asia		3	9.7%
	Central America		2	6.5%
	Eastern Europe		2	6.5%
	South-eastern Asia		2	6.5%
	Southern Europe		1	3.2%
Medical Education Status	Graduate of Medical School		21	67.7%
	3rd-year Student (= 1st-year in the U.S.)		1	3.2%
	4th-year Student (= 2nd-year in the U.S.)		4	12.9%
	5th-year Student (= 3rd-year in the U.S.)		2	6.5%
	6th-year Student (= 4th-year in the U.S.)		3	9.7%
Training Status	Graduate		25	80.6%
	In School		6	19.4%
	Years since graduation, mean±SD (range)		5.54±6.04 (0-27)	
United States Clinical Experience (USCE)	Yes		18	58.1%
	Month of USCE, mean±SD		3.29±7.40	
Region of Medical School^**^	Eastern Asia		13	41.9%
	Caribbean		5	16.1%
	Western Asia		3	9.7%
	South-eastern Asia		3	9.7%
	Southern Asia		2	6.5%
	Central America		2	6.5%
	Eastern Europe		2	6.5%
	Southern Europe		1	3.2%
USMLE	Step 1 Pass		25	80.6%
	Step 2 Pass		14	45.2%
	Step 3 Pass		1	3.2%
Research	Yes		15	48.4%
	No		14	45.2%
	Prefer Not to Say		2	6.5%
Type of Research	Abstract or Poster	0-5	25	80.6%
		6-10	2	6.5%
		11-15	1	3.2%
		Prefer Not to Say	3	9.7%
	Publication (Submitted or In-Print)	0-5	26	83.9%
		6-10	1	3.2%
		11-15	1	3.2%
		Prefer Not to Say	3	9.7%
	Total		31	100.0%

In terms of educational status, most of the participants (67.7%) have already graduated, while about one-third (32.3%) were in their third to sixth year of medical school training (equivalent to first to fourth year in a four-year post-bachelor medical school). Among graduates, the average time since graduation was 5.5 years (95%CI: 2.99-8.09), ranging from 0 to 27 years. A majority had passed USMLE Step 1 (80.6%), with smaller proportions completing Step 2 (45.2%) and Step 3 (3.2%). More than half of the attendees (58.1%) reported US clinical experience, averaging 3.29 months (95%CI: 0.17-6.42). Nearly half (48.4%) engaged in research, most with 0-5 abstracts or posters (80.6%) and 0-5 publications (83.9%).

Survey outcomes

**Figure 1:**
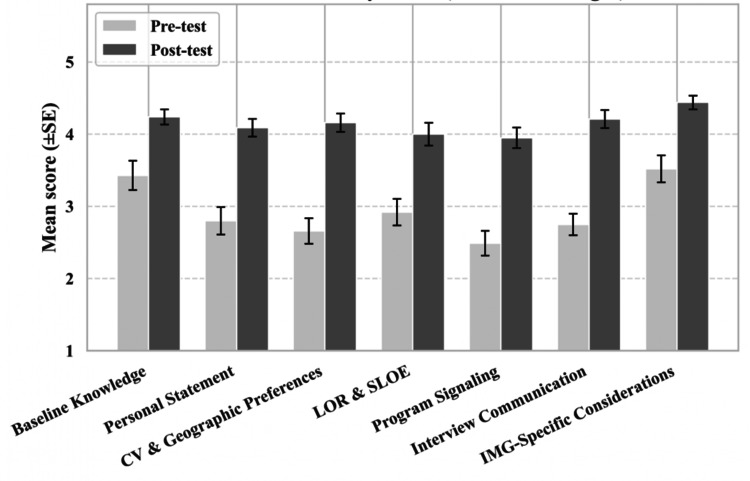
Bar Chart Comparing Pre- and Post-Survey Scores Among All Seven Domains (N = 31) Bars represent mean scores ± standard error (SE) for pre-test and post-test responses across the seven survey domains (N = 31). Higher scores indicate greater perceived understanding or preparedness within each domain. SE: standard error, PS: personal statement, CV: curriculum vitae, LOR: letter of recommendation, SLOE: standardized letter of evaluation, IMG: international medical graduate

**Figure 2:**
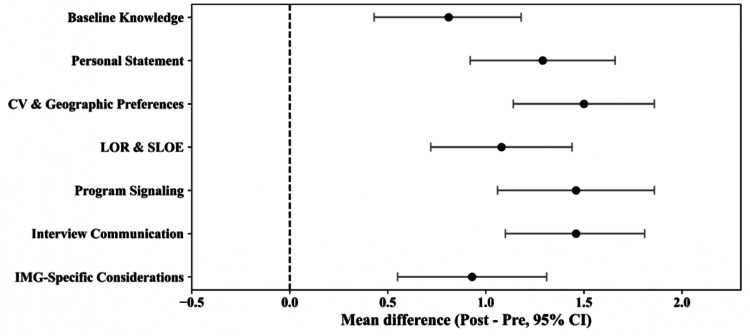
Forest Plot Demonstrating Improvement of Score Among Seven Domains (N = 31) Points represent the mean difference between post-test and pre-test scores, and horizontal lines indicate the 95% confidence intervals (CI) for each domain (N = 31). The vertical dashed line at 0 indicates no change between pre- and post-survey scores. Positive values indicate improvement following the panel discussion. CI: confidence interval, PS: personal statement, CV: curriculum vitae, LOR: letter of recommendation, SLOE: standardized letter of evaluation, IMG: international medical graduate

Reliability testing indicated strong internal consistency, with Cronbach’s alphas more than 0.70 for all domains and an overall score of 0.96 (Table 2). Significant improvements across all seven domains were observed from pre- to post-panel (Figures 1, 2).

**Table 2:** Reliability Analysis Using Cronbach’s Alpha (N = 31) Cronbach’s α values were calculated to evaluate the internal consistency of survey items within each domain. Item Number indicates the number of questionnaire items included in each domain. α (ALL) represents the Cronbach’s alpha coefficient calculated across all items within the domain. A Cronbach’s alpha value ≥ 0.70 was considered indicative of acceptable internal consistency. α: Cronbach’s alpha, PS: personal statement, CV: curriculum vitae, LOR: letter of recommendation, SLOE: standardized letter of evaluation, IMG: international medical graduate

Domain	Item Number	α (ALL)
1. Baseline understanding about PM&R	3	0.796
2. PS	4	0.881
3. CV & Geographic preferences	4	0.867
4. LOR & SLOE	4	0.865
5. Program signaling	4	0.842
6. Interview & Post-interview communication	4	0.79
7. IMG-specific considerations	4	0.819
All Domain	27	0.96
Satisfaction	6	0.915

Tests of normality indicated non-normal distributions for baseline knowledge (Domain (1)) and IMG-specific considerations (Domain (7)), while Domains (2) to (6) met normality assumptions (Tables 3, 4). 

**Table 3:** Normality of Seven Domains (N = 31) The Shapiro–Wilk test was used to assess the normality of score distributions for each domain (N = 31). Statistic represents the Shapiro–Wilk test statistic (W), df indicates the number of observations, and Sig. represents the p-value for the normality test. A p-value < 0.05 was considered indicative of deviation from normality. PS: personal statement, CV: curriculum vitae, LOR: letter of recommendation, SLOE: standardized letter of evaluation, IMG: international medical graduate

Tests of Normality	Shapiro-Wilk		
	Statistic (W)	df	Sig.
1. Baseline understanding about PM&R	0.89	31	0.004
2. PS	0.962	31	0.337
3. CV & Geographic preferences	0.978	31	0.747
4. LOR & SLOE	0.97	31	0.508
5. Program signaling	0.963	31	0.356
6. Interview & Post-interview communication	0.95	31	0.159
7. IMG-specific considerations	0.906	31	0.01

**Table 4:** Survey Outcome for Domains without Normal Distribution (N = 31) Pre-test and post-test mean scores are presented for domains that did not meet the normality assumption based on the Shapiro–Wilk test. Mean Difference represents the change in score between post-test and pre-test values. SD indicates the standard deviation of the score differences. P (Holm) represents the p-value adjusted using the Holm correction for multiple comparisons. A p-value < 0.05 was considered statistically significant. SD: standard deviation, PM&R: physical medicine and rehabilitation, IMG: international medical graduate.

Item	Pre-test Mean	Post-test Mean	Mean Difference	SD	P (Holm)
1. Baseline understanding about PM&R	3.43	4.24	0.81	1.01	<0.05
7. IMG-specific considerations	3.52	4.44	0.93	1.04	<0.05

Following correction for multiple significant analyses, all domains showed significant improvement (adjusted p < 0.01) (Table 5). The largest gains occurred in CV and geographic preferences, program signaling, and interview or post-interview communication. More modest but noteworthy increases were seen in personal statement and IMG-specific considerations. Understanding of the baseline knowledge about PM&R, LOR, and SLOE had also increased significantly.

**Table 5:** Survey Outcomes with Average Scores among All Seven Domains in Details (N = 31) Pre-test and post-test mean scores are presented for each survey item across the seven domains (N = 31). Mean Difference represents the change in score between post-test and pre-test values. SD indicates the standard deviation of the score differences. P (Holm) represents the p-value adjusted using the Holm correction for multiple comparisons. A p-value < 0.05 was considered statistically significant. SD: standard deviation, PM&R: physical medicine and rehabilitation, PS: personal statement, CV: curriculum vitae, LOR: letter of recommendation, SLOE: standardized letter of evaluation, IMG: international medical graduate, ERAS: Electronic Residency Application Service

Item	Pre-test Mean	Post-test Mean	Mean Difference	SD	P (Holm)
1. Baseline understanding about PM&R					
A1. Core concepts of PM&R	3.77	4.32	0.55	1.21	<0.01
A2. Process of applying to PM&R	3.35	4.32	0.97	1.20	<0.01
A3. Preparing/submitting ERAS	3.16	4.06	0.90	1.25	<0.01
A Average	3.43	4.24	0.81	1.01	<0.01
2. PS					
B1. Understand what PM&R programs look for in a PS	2.84	4.32	1.48	1.26	<0.01
B2. Know which resources to use for a PS	2.68	3.87	1.19	1.25	<0.01
B3. Confident in writing a PS reflecting strengths/goals	3.06	4.10	1.03	1.17	<0.01
B4. Confident in strategically revising a PS	2.61	4.06	1.45	1.03	<0.01
B Average	2.80	4.09	1.29	1.00	<0.01
3. CV & Geographic preferences					
C1. Structure & strengthen CV	2.68	4.13	1.45	1.09	<0.01
C2. Indicate geographic preferences	2.48	4.16	1.68	1.05	<0.01
C3. Select activities to highlight strengths	2.90	4.23	1.32	1.25	<0.01
C4. Choose preferred regions	2.58	4.13	1.55	1.21	<0.01
C Average	2.66	4.16	1.50	0.99	<0.01
4. LOR & SLOE					
D1. Difference between LOR and SLOE	3.00	4.06	1.07	1.15	<0.01
D2. Appropriate letters to ask	3.00	4.26	1.26	0.97	<0.01
D3. Confident to request a strong LOR	3.06	3.97	0.90	1.25	<0.01
D4. Confident to choose appropriate letters	2.61	3.71	1.10	1.35	<0.01
D Average	2.92	4.00	1.08	0.98	<0.01
5. Program signaling					
E1. Understand the signaling system	2.48	4.00	1.52	1.15	<0.01
E2. Strategically signal programs	2.26	3.87	1.61	1.36	<0.01
E3. Manage increased signals	2.61	4.06	1.45	1.26	<0.01
E4. Choose programs to signal	2.61	3.87	1.26	1.24	<0.01
E Average	2.49	3.95	1.46	1.09	<0.01
6. Interview & Post-interview communication					
F1. Know what to expect in interviews	2.74	4.23	1.48	1.06	<0.01
F2. Second looks & post-interview communication	2.52	4.06	1.55	1.26	<0.01
F3. Confident to present strengths	2.90	4.23	1.32	1.11	<0.01
F4. Confident to communicate after interviews	2.84	3.87	1.03	1.11	<0.01
F Average	2.75	4.21	1.46	0.96	<0.01
7. IMG-specific considerations					
G1. IMG challenges & strengths	3.48	4.45	0.97	1.22	<0.01
G2. Differences in systems	3.61	4.52	0.90	1.17	<0.01
G3. Confidence in navigating the match	3.10	4.23	1.13	1.28	<0.01
G4. Resilience in the IMG process	3.87	4.58	0.71	1.30	<0.01
G Average	3.52	4.44	0.93	1.04	<0.01

Satisfaction

**Figure 3:**
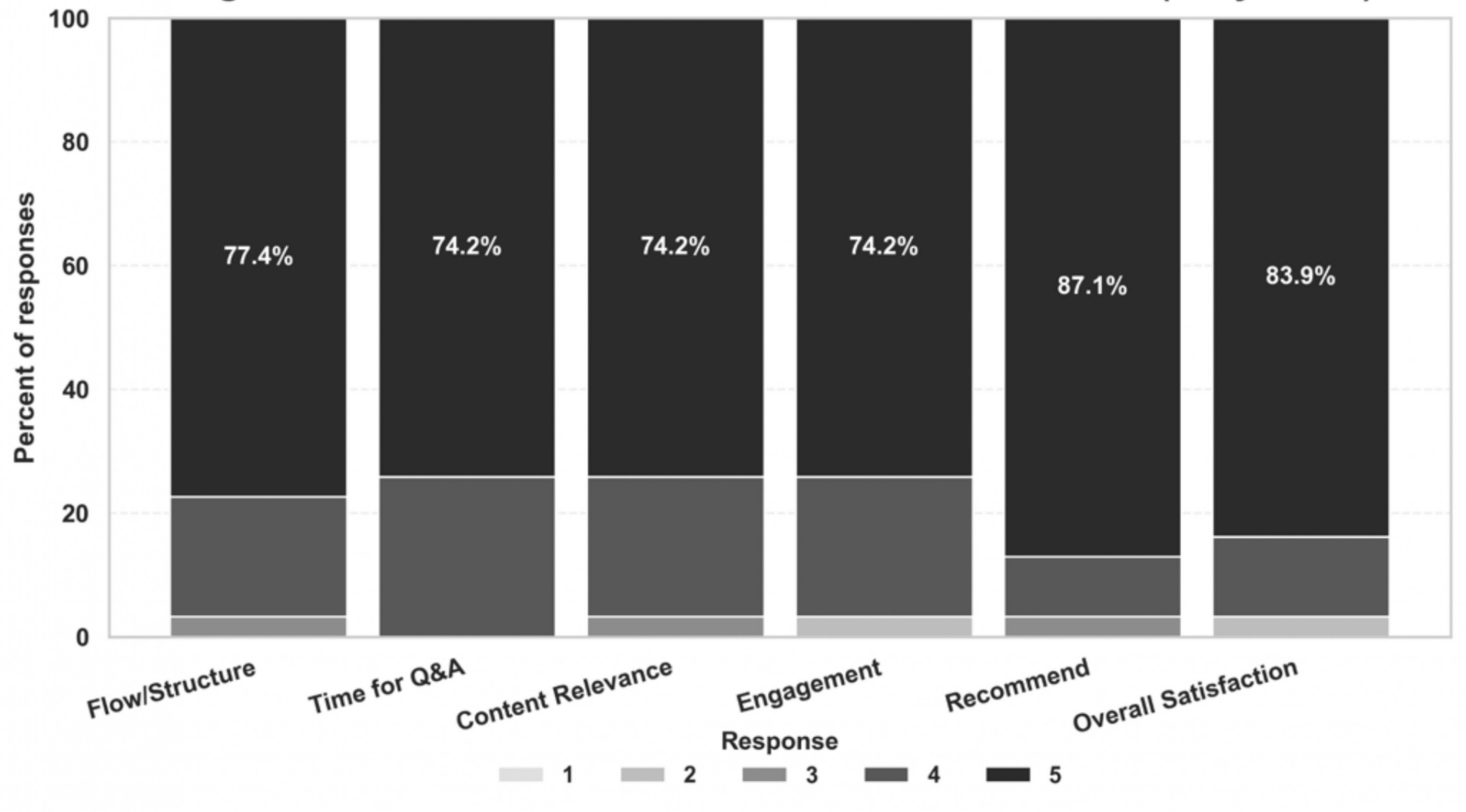
Stacked Bar Chart for Satisfaction (N = 31) Stacked bars represent the percentage distribution of responses for each satisfaction item (N = 31). Responses were measured using a 5-point Likert scale (1 = strongly disagree, 5 = strongly agree). Percentages shown within the bars indicate the proportion of respondents selecting higher agreement levels (scores of 4 or 5).

Satisfaction ratings were consistently high (Table 6). Mean scores across the six items ranged from 4.68 to 4.84 out of five, each substantially exceeding the neutral midpoint of three (p < 0.01). Overall satisfaction was rated at 4.77 (Figure 3). Respondents strongly endorsed the webinar as well-structured, time-appropriate, relevant, engaging, and worth recommending to peers, with p-values all less than 0.01.

**Table 6:** Satisfaction (N = 31) Mean satisfaction scores are presented for six evaluation items based on a 5-point Likert scale (1 = strongly disagree, 5 = strongly agree). SD represents the standard deviation of the responses. P* represents the p-value from a one-sample test comparing the mean score to the neutral midpoint of the scale (3). A p-value < 0.05 was considered statistically significant. SD: standard deviation, IMG: international medical graduate * Compared to the median of 3

Item	Mean	SD	P^*^
1. Flow and structure	4.74	0.514	<0.01
2. Time for discussion	4.74	0.445	<0.01
3. Content relevance	4.71	0.529	<0.01
4. Moderators engaging	4.68	0.653	<0.01
5. Recommend to IMG	4.84	0.454	<0.01
6. Overall satisfaction	4.77	0.617	<0.01

## Discussion

This PD panel represents the first initiative of its kind, aiming to strengthen IMGs’ understanding of the PM&R residency application process. By including IMGs from a wide range of cultural and educational backgrounds, this initiative underscores the importance of diversity, equity, and inclusion in PM&R. As shown by the results, IMGs reported a significant improvement in self-perceived knowledge and confidence across all seven key application domains, highlighting the value of direct PD engagement in IMG-focused education.

Baseline knowledge of PM&R

IMGs demonstrated statistically significant improvement in their baseline knowledge of PM&R after attending this PD panel webinar. As shown by the results, the average reported increase on a five-point Likert scale between pre- and post-session scores was 0.81 points, highlighting the importance of educational panels in improving IMGs’ awareness and exposure to PM&R.

While the field of PM&R continues to gain recognition, less than half of US medical schools are associated with PM&R programs, contributing to the field’s growing competitiveness [7]. For IMGs, this challenge is further compounded by limited global exposure to PM&R, which creates additional barriers to acquiring appropriate experience in the field. This has been widely attributed to the absence of uniform integration of PM&R into international medical school curricula. The study by Al-Washmi et al. on medical students in Saudi Arabia demonstrated their knowledge of PM&R as low to moderate [8]. Moreover, a study conducted in Singapore revealed that, after only a one-week PM&R clerkship, 98.6% of students had improved scores on their knowledge of PM&R [9]. Consequently, as limitations to PM&R exposure around the world remain significant, it is important to assess the impact of such educational webinars on IMGs’ baseline knowledge of the field.

Writing PSs, structuring CVs, and geographic preferences

Remarkably, the analysis demonstrated a statistically significant improvement in the self-perceived confidence and knowledge of IMGs in writing PSs, structuring CVs, and navigating geographical preferences. The webinar included a set of questions tailored to better understand what PM&R program directors look for in the PS and CV of IMGs, and for advice on determining a geographical preference. Accordingly, the results showed a 1.29-point increase in PS scores on a five-point Likert scale; moreover, the greatest change in mean difference between pre- and post-session scores across all seven domains was observed in CV preparation and geographic preference, demonstrated by a 1.50-point increase on a five-point Likert scale.

Two of the most important components of the medical student's application for the US residency process are the PS and CV [10]. The PS gives program directors a sense of who the applicant is, while the CV lists an applicant's accomplishments, especially during medical school [11]. Limited information is available to guide IMGs on how to formulate their PS and structure their CV when applying to PM&R. Accordingly, it is important to understand the limitations that IMGs encounter when preparing for these key elements of the residency application.

A more recent addition to the residency application process enables Match applicants to decide on a geographic preference. Ultimately, this allows applicants to provide region-specific details, enabling them to indicate their geographic preference in up to three regionally grouped states, as well as a preference for urban or rural settings [11]. This has further added to the complexity of the application process, as PDs from participating specialties in the 2022 Match Cycle widely considered geographic preferencing useful when deciding between applicants [11]. Similarly, limited information is available to guide IMGs on how to navigate geographical preference considerations; accordingly, as the specialty continues to attract more IMGs to US residency programs, there is a growing need for exposure to practical strategies and insights for successfully navigating the PM&R application process [1,2].

LOR and SLOE

This panel further strengthened IMGs’ understanding of requesting LORs and SLOEs. Post-session survey data showed a 1.08-point increase on a five-point Likert scale in participants’ self-reported knowledge and confidence regarding the process, representing a statistically significant improvement.

LORs are a central element of an applicant's portfolio for the US Match process. They convey an applicant's potential for success, and are important in highlighting applicant qualities often missed or unrecognized in CVs, test scores, and grades [12]. Unfortunately, previous studies have demonstrated that IMGs have less supportive and shorter letters of recommendation when compared to US medical graduates [13]. While the reasons remain undetermined, one possible explanation may be IMGs' unfamiliarity with the process of requesting LORs from the appropriate mentors, and at the optimal time, as most IMGs have minimal exposure to the US medical education system.

Moreover, SLOEs are becoming more widely incorporated into the residency application process to improve the objectivity of LORs [14]. As a new addition to the PM&R application process, limited literature and guidance are available, consequently adding to the complexity of the US residency application process for IMGs.

Program signaling

This webinar demonstrated significant improvement in IMGs’ self-perceived confidence and knowledge of the program signaling process. As shown by the results, this domain had the lowest pre-session mean among all categories, with a score of 2.49 on a five-point Likert scale. On the other hand, this category had the second-highest increase in post-session mean (1.46), demonstrating the importance of such panels in improving IMG knowledge of the residency application process.

The preference signaling system (program signaling system), although relatively recent, became the most important aspect of the ERAS Supplemental Application and was later integrated into the main ERAS application [15,16]. Fundamentally, program signaling provides applicants with a more standardized method to inform PDs of interest in their specific residency programs [11]. The number of program signals available to applicants in PM&R has increased to 20 for the 2026 application cycle [17]. Accordingly, this provides applicants with a heightened responsibility of precisely choosing the programs they find most fit for them. As discussed by Alkhouri et al., the novelty of program signaling has left many applicants uncertain about how to use signals effectively, highlighting the need for improved applicant education [18].

Interview and post-interview communication

This study further demonstrated significant improvements in IMGs’ confidence and knowledge in interview and post-interview communication. As shown by the results, the mean difference between pre- and post-session scores had a 1.46 increase on a five-point Likert scale.

Interview experiences and post-interview communications can be a great source of distress for applicants [18,19]. Moreover, there is a limited understanding of what forms of communication are effective and acceptable by PDs outside of the interview day [18]. As shown by Donaldson et al, formal residency interview training programs can meaningfully improve applicants’ confidence after structured mentorship [20]. While this educational webinar was not a formal one-on-one residency interview mentoring session, it did emphasize key elements of interview preparation and professional communication that are important to a successful residency application. Accordingly, while the webinar shared the perspectives of PM&R PDs on navigating residency interviews and identifying acceptable forms of post-interview communication for IMG applicants, further education is needed to promote long-term improvement in scores.

IMG-specific considerations

This category also showed notable improvements in IMGs’ confidence and knowledge, with a 0.93-point increase on a five-point Likert scale, underscoring the effectiveness of educational webinars in addressing IMG-specific challenges.

As shared by Zepeda et al., the challenges confronted by IMGs begin well before the residency application, as they must build a highly competitive profile to qualify for a chance of matching [21]. To further understand the challenges that IMGs face and how to overcome them, this IMG-specific section was dedicated to further empowering IMGs navigating the PM&R application process. The questions focused on several key topics, including common concerns that programs have regarding IMGs, visa sponsorship and status, exam scores and potential cutoffs, and additional issues raised by the attendees throughout the session.

Participant satisfaction

As shown by the results, satisfaction ratings were consistently high, with an overall post-session satisfaction rating of 4.77. Accordingly, attendees strongly endorsed the webinar as well-structured, relevant, engaging, and worth recommending to peers interested in the field.

Web-based educational activities have previously shown high levels of satisfaction among healthcare providers [22]. Although limited literature directly addresses PM&R residency panels, studies of educational workshops in medical education using Kirkpatrick’s model show that high participant satisfaction is strongly associated with improved learning transfer and later application of acquired skills [23].

Limitations

IMGs applying to the US Match process have historically faced considerable challenges, including, but not limited to, language and communication barriers, clinical and educational exposure, work culture, and discrimination. Moreover, limited data is available on strategies to support IMGs’ transitioning into their host countries [24]. While the panel focused on strategies to strengthen IMGs’ knowledge and confidence in navigating the residency application process, these broader challenges were not within the scope of discussion and should be kept in mind when considering the implications of the findings.

This study is also limited by the extent to which information from a single one-hour virtual panel webinar can be retained. Kehoe et al. explain that educational interventions, including induction programs and training workshops, are generally helpful, but not sustained long enough to ensure the transfer and retention of knowledge and skills [25].

Further limitations include the session’s sample size, participating audience, and reliance on objective assessments for measuring the accuracy of the results. The relatively small sample size (31 paired responses) restricts the generalizability of findings and may not fully represent the broader IMG population. Moreover, most participants who attended the panel were of East Asian origin; this likely reflects recruitment efforts concentrated mostly on East Asian networks, which may have introduced selection bias. Although exact proportions from these countries are not specified in national IMG data, broader trends show that India serves as the primary country of origin for the highest proportion of IMGs, contributing to nearly 23% of the total IMG count currently practising in the US [26]. Other data demonstrates that South‑Central and South‑Eastern Asia contribute to nearly 19.9% and 9.1% of IMG training origins in Family Medicine, respectively [27]. This data does not align with the audience, as around 35% were from East Asia alone. Moreover, outcomes relied on self-reported knowledge and confidence rather than objective assessments or actual match outcomes, which may limit the accuracy of the measured results.

## Conclusions

This pilot study demonstrated significant improvements in the knowledge and confidence of IMGs in the PM&R residency application process after a PD-led panel, with the highest gains in CV and geographic preference, program signaling, and interview communications. High satisfaction scores also support the potential feasibility of this panel and may be helpful in improving the diversity of the workforce in PM&R. Future research should further evaluate match success rates among IMGs across upcoming match cycles, while also examining trends in the growing number and geographic diversity of IMG applicants.

## Appendices

**Table 7:** Questionnaire for Panel Attendees, Including Seven Domains and 27 Items Domains (1) to (7) were answered using a Likert scale, with one indicating "Strongly disagree" and five indicating "Strongly agree".

Domains	Questions in pre- and post-surveys
Background information	Gender
	Age
	Race
	Citizenship
	Medical education status
	Year of graduation (YOG)
	United States Clinical Experiences (USCE) (Yes / No)
	Region of medical school
	USMLE STEP 1 results (Pass / Fail)
	USMLE STEP 2 results (Pass / Fail)
	USMLE STEP 3 results (Pass / Fail)
	Past research experiences (Yes / No)
	Type and number of past research (Abstract or poster / Publication, submitted or in-print)
(1) Baseline understanding about PM&R	I am familiar with the core concepts of PM&R.
	I am familiar with the overall process of applying to PM&R residency programs in the U.S.
	I am familiar with how to prepare and submit a residency application through the Electronic Residency Application Service (ERAS).
(2) Writing a PS	I understand what PM&R residency programs look for in a PS.
	I know which resources to use to write an effective PS.
	I am confident that I can write a PS that reflects my unique strengths and goals.
	I am confident that I can strategically revise my PS.
(3) Writing a CV and arranging geographic preferences	I know how to structure and strengthen my CV, including the most meaningful experiences, as an IMG applicant to PM&R.
	I understand how to indicate geographic preferences and their impact on the ERAS supplemental application.
	I feel confident in selecting activities that best highlight my strengths for PM&R.
	I can effectively choose preferred regions based on my needs when applying to PM&R programs.
(4) Communication for LOR and SLOE	I understand the difference between a LOR and a SLOE.
	I understand how many letters are required and who would be most appropriate to ask for letters.
	I am confident that I can approach and request a strong LOR or SLOE from a physiatrist.
	I am confident in my ability to choose the most appropriate combination of LORs and SLOEs to submit.
(5) Utilization of program signaling	I understand how the signaling system works in the PM&R residency application process.
	I know how to strategically signal PM&R programs that align with my fit.
	I am aware of the increase in signals (from 8 to 20) and confident in my ability to manage them effectively for PM&R residency programs.
	I can choose which programs to signal based on my competitiveness and preferences.
(6) Interview & post-interview communication	I know what to expect from interview invitations and how to respond appropriately.
	I understand how to approach second looks and post-interview communication in a professional manner.
	I am confident in my ability to present my strengths effectively during interviews.
	I am confident in my ability to communicate professionally with programs after interviews.
(7) Other IMG-specific considerations	I understand the unique challenges and strengths that IMGs bring to the PM&R match process.
	I understand the differences between foreign medical systems and the U.S. system.
	I am confident that I can successfully navigate the PM&R match process as an IMG.
	I can remain resilient when facing common challenges as an IMG in the U.S. residency application process.
